# Evaluation of peripapillary retinal nerve fiber layer thickness in intracranial atherosclerotic stenosis

**DOI:** 10.1186/s12886-023-03196-6

**Published:** 2023-11-13

**Authors:** Yuan Gao, Xuxiang Zhang, Di Wu, Chuanjie Wu, Changhong Ren, Tingting Meng, Xunming Ji

**Affiliations:** 1 Department of Biomedical Engineering, School of Biological Science and Medical Engineering, Beihang University, 100191 Beijing, China; 2 Department of Ophthalmology, Xuanwu hospital, Capital Medical University, 100053 Beijing, China; 3 China-America Institute of Neuroscience, Xuanwu hospital, Capital Medical University, 100053 Beijing, China; 4 Department of Neurology, Xuanwu hospital, Capital Medical University, 100053 Beijing, China; 5 Beijing Key Laboratory of Hypoxic Conditioning Translational Medicine, Xuanwu hospital, Capital Medical University, 100053 Beijing, China

**Keywords:** Stroke, Intracranial atherosclerotic stenosis, Retinal nerve fiber layer

## Abstract

**Purpose:**

To evaluate the peripapillary retinal nerve fiber layer thickness (pRNFL) in patients with intracranial atherosclerotic stenosis (ICAS).

**Methods:**

A cross-sectional study was performed in a general hospital. The intracranial atherosclerotic stenosis was evaluated by digital subtraction angiography (DSA), computed tomography angiography (CTA) or magnetic resonance angiography (MRA). High-definition optical coherence tomography (HD-OCT) was used to evaluate the peripapillary retinal nerve fiber layer thickness.

**Results:**

A total of 102 patients, including 59(57.8%) patients with ICAS and 43(42.2%) patients without ICAS, were finally analysed in the study. The peripapillary retinal nerve fiber layer thickness (pRNFL) was reduced significantly in the average, the superior and the inferior quadrants of the ipsilateral eyes and in the superior quadrant of the contralateral eyes in patients with ICAS compared with patients without ICAS. After multivariate analysis, only the superior pRNFL thickness in the ipsilateral eyes was significantly associated with ICAS (OR,0.968; 95% CI,0.946–0.991; *p* = 0.006). The area under receiver operator curve was 0.679 (95% CI,0.576–0.782) for it to identify the presence of ICAS. The cut-off value of the superior pRNFL was 109.5 μm, and the sensitivity and specificity were 50.8% and 83.7%, respectively.

**Conclusion:**

The superior pRNFL in the ipsilateral eye was significantly associated with ICAS in this study. Larger studies are needed to explore the relation between pRNFL and ICAS further.

## Introduction

The retina shares in the same embryological origin, anatomic features, blood barriers, and physiological properties with the brain [1]. The retinal nerve fiber layer (RNFL), which is made up of unmyelinated axons derived from retinal ganglion cells (RGCs), is the only structure in the central nervous system where naked axons can be examined in vivo [2]. Therefore, the retinal nerve fiber layer thickness might be a promising parameter for monitoring axonal loss in ocular and neurological diseases [3, 4]. Studies in animal models have indicated that ischemic stroke induced by middle cerebral artery occlusion (MCAO), may lead to retinal functional impairment, particularly retinal layer thinning [1, 5, 6]. In addition, some clinical studies also demonstrated that retinal nerve fiber layer thickness was reduced in patients with cerebral infarction [7, 8]. Thus, strokes may not only affect the visual pathway leading to homonymous defects but also cause retinal nerve damage [9]. As a major cause of stroke, intracranial atherosclerosis stenosis (ICAS) was associated with more severe symptoms, a longer-stay in the hospital and high risk of recurrent subclinical, clinical ischemic events and high rate of stroke mortality, compared with the other stroke subtypes [10, 11]. The main mechanisms of ischemic stroke due to ICAS include artery-to-artery embolism or occlusion of the artery resulting in plaque rupture with in-situ thrombosis and hemodynamic damage due to highly stenotic plaques [12]. The visual pathway is mainly supplied by the ophthalmic and intracranial arteries [9]. However, it is still unknown whether RNFL thickness differs in patients with and without ICAS. Here, we aim to evaluate the peripapillary retinal nerve fiber layer thickness in patients with intracranial atherosclerotic stenosis.

## Methods

### Study design and participants

The cross-sectional, retrospective study was performed at ophthalmic consultation center in Xuanwu hospital of Capital Medical University between January 1,2019 and February 20,2022. The inclusion criteria: (1) age above 30 years; (2) had digital subtraction angiography (DSA), computed tomography angiography (CTA) or magnetic resonance angiography (MRA) and optical coherence tomography (OCT) examinations at the same time were enrolled consecutively; (3) had a TIA, ischemic stroke or hemorrhagic stroke diagnosed based on magnetic resonance imaging of brain within 14 days. We excluded patients with non-atherosclerotic stenosis, such as arteritis, moyamoya disease and arterial dissection; patients with multiple sclerosis, intracranial mass lesions and venous sinus thrombosis; patients with ocular diseases including pathologic myopia, mature cataract, glaucoma, macular diseases, optic neuritis, optic neuropathy and have a history of posterior segment trauma and retinal surgery. This study was carried in accordance with the Declaration of Helsinki and performed with a waiver of informed consent under the approval of the Ethics Committee of Xuanwu Hospital, Capital Medical University (No.2,022,101).

### Assessment of intracranial atherosclerotic stenosis

The major intracranial large arteries including the anterior cerebral artery (ACA), middle cerebral artery (MCA), posterior cerebral artery (PCA), the intracranial segment of internal carotid artery (ICA), the intracranial segment of vertebral artery (VA), and basilar artery (BA) [13, 14] were evaluated by neuroimaging examinations such as DSA, MRA or CTA. Degrees of stenosis were calculated by referring to the (Warfarin-Aspirin Symptomatic Intracranial Disease) WASID study in which the equation was as follows: “percent stenosis = [(1-(D_stenosis_/D_normal_))] ×100, where D_stenosis_ = the diameter of the artery at the site of the most severe degree of stenosis and D_normal_= the diameter of the proximal normal artery”. [15]. According to the severity of stenosis and the imaging appearance of atherosclerosis, we defined the vessels with ≥ 50% stenosis or occlusion as presence of intracranial atherosclerosis stenosis [11].

### Optical coherence tomography (OCT)

All patients were conducted with high-definition OCT (HD-OCT, Cirrus 5000, Carl Zeiss Meditec, software version 9.5.2, CA, USA) by a single trained, experienced technician masked to the patient’s information. Peripapillary RNFL thickness was measured by using optic disk 200 × 200 protocol with a custom 1.73 mm ring centered at the papilla. Using the built-in software, the retinal thickness deviation map was generated automatically. The four RNFL quadrants: superior (S), inferior (I), temporal (T), and nasal (N) and average thickness of RNFL were calculated for each eye. We defined the eyes on the same side of the major intracranial arteries stenosis as the ipsilateral eyes and on the opposite side as the contralateral eyes.

### Other variables

The following demographic and clinical variables of patients from the electronic medical records were used for this study: age, gender, body mass index(BMI), hypertension, diabetes mellitus, hyperlipidemia, atrial fibrillation, myocardial infarction, coronary artery disease, smoking, alcohol use, family history of stroke, systolic blood pressure, diastolic blood pressure, and biochemical testing of serum including glycosylated hemoglobin, glucose, total cholesterol, triglycerides, high density lipoprotein cholesterol, low-density lipoprotein cholesterol, uric acid and homocysteine. The treatment measures including antiplatelet drugs, lipid-lowing drugs and endovascular treatment were also recorded.

### Statistical analysis

Data were described as mean ± SD, median and interquartile range (IQR), or frequency counts and percent. Nonparametric variables were used to compared with two groups by using Mann-Whitney U test. Student’s t-test was used to compare with means of continuous variables which conform to normality (determined by the Shapiro-Wilk test). Spearman’s rank correlation coefficient was used to assess the correlation between the retinal nerve fiber layer thickness and intracranial atherosclerotic stenosis. Binary logistic regression was used to examine variables that were significant in univariate analysis. The area under receiver operating characteristic (ROC) curves was used to assess the diagnostic values of the retinal nerve fiber layer thickness and the cut-off value was determined according to the maximum Yonden index. The missing value of baseline characteristics was less than 5% and filled by mean value in this study. The sensitivity analysis showed that there is no significant difference between imputing and excluding the missing value. Significance level was set to α = 0.05. All tests were 2 sided. Statistical analyses were conducted using SPSS, version 25.0 (IBM Corporation).

## Results

### Patient characteristics

**Fig. 1:**
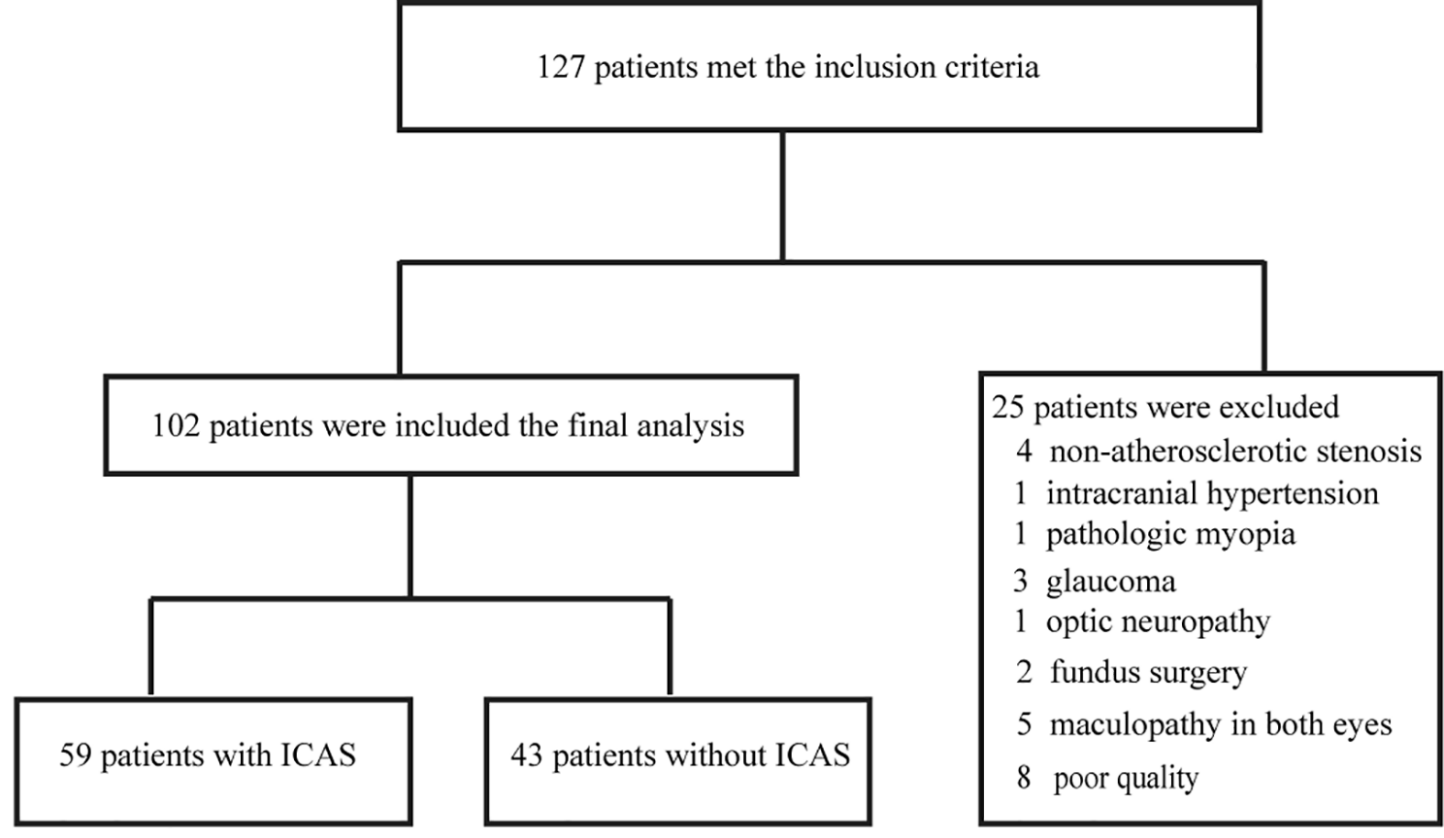
Study-flow chart

A total of 127 patients were assessed for eligibility. Finally, 102 patients (median age,58; Men,79.4%) were included in this study. Of 102 patients, 59 (57.8%) patients were diagnosed with ICAS and 43 (42.2%) patients without ICAS (Fig. 1). The baseline demographic characteristics were summarized in Table 1. Patients with ICAS or not had similar age, gender composition and body mass index (*p* > 0.05 each). The prevalence of hypertension and diabetes were also similar in patients with ICAS or not. 93 patients (91.2%) had an ischemic stroke, 16 patients (15.7%) had a TIA, 7 patients (6.9%) had complications of TIA and ischemic stroke, and 2 patients (2.0%) had complications of stroke including cerebral infarction and cerebral hemorrhage. No differences have been found in the cerebrovascular events including TIA, ischemic stroke or hemorrhagic stroke in patients with ICAS or not (*p* > 0.05 each). In ischemic stroke etiologic subtypes which according to Trial of Org 10,172 in Acute Stroke Treatment (TOAST) criteria, large-artery atherosclerosis was accounted for the largest proportion (63 of 93 patients, 67.7%) when compared with other etiologies. Patients with ICAS were more likely to have large-artery atherosclerosis stroke subtype than those without ICAS (83.3% vs. 46.2%, *p* < 0.001). There were no differences in treatments choices between patients with and without ICAS, including antiplatelet drugs, lipid-lowing drugs and endovascular treatment. In addition, there were no significant differences between patients with and without ICAS in other variables(*p* > 0.05 each).

**Table 1:** Baseline characteristics

Characteristic	All n = 102	With ICAS n = 59	Without ICAS n = 43	*p* value
**Baseline**				
Age, median, IQR, years	58(43-65.3)	58 (47–66)	58(40–65)	0.611
Gender				
Male, n (%)	81(79.4)	49(83.1)	32(74.4)	0.287
Female, n (%)	21(20.6)	10(16.9)	11(25.6)	
Body mass index, median, IQR, kg/m2	26(23.4–28.1)	26(23.4–28.3)	25.6(23.4–27.7)	0.932
Hypertension, n (%)	69(67.6)	42(71.2)	27(62.8)	0.371
Diabetes mellitus, n (%)	28(27.5)	20(33.9)	8(18.6)	0.087
Hyperlipidemia, n (%)	41(40.2)	26(44.1)	15(34.9)	0.350
Atrial fibrillation, n (%)	3(2.9)	2(3.4)	1(2.3)	0.753
Myocardial infarction, n (%)	5(4.9)	1(1.7)	4(9.3)	0.079
Coronary artery disease, n (%)	20(19.6)	10(16.9)	10(23.3)	0.428
Alcohol use, n (%)	48(47.1)	30(50.8)	18(41.9)	0.369
Smoking, n (%)	57(55.9)	35(59.3)	22(51.2)	0.412
Family history of stroke, n (%)	25(24.5)	16(27.1)	9(20.9)	0.473
Systolic blood pressure, median (IQR)	138(125.8-153.3)	140(130–155)	134(120–153)	0.075
Diastolic blood pressure, median (IQR)	85.5(78–95)	87(79–95)	82(76–94)	0.360
Glycated hemoglobin, median (IQR)	5.8(5.3–6.3)	5.9(5.5–6.5)	5.6(5.3–6.2)	0.070
Glucose, median (IQR)	4.9(4.4–5.7)	5.1(4.4–6.4)	4.9(4.4–5.2)	0.111
Triglyceride, median (IQR)	1.3(0.9–1.6)	1.2(0.9–1.6)	1.3(0.9–1.9)	0.454
Total cholesterol, median (IQR)	3.8(3.1–4.5)	3.6(3.1–4.2)	4.1(3.2–4.7)	0.128
High-density lipoprotein, median (IQR)	1.0(0.8–1.1)	1.0(0.8–1.1)	1.0(0.8–1.1)	0.812
Low-density lipoprotein, median (IQR)	2.2(1.6–2.8)	2.1(1.6–2.5)	2.3(1.6-3.0)	0.255
Uric acid, mean (SD)	327.2 (76.8)	316.9(79.3)	341.4(71.6)	0.234
Homocysteine, median (IQR)	14.2(11.5–17.4)	14.4(12.2–17.4)	13.1(11-17.1)	0.166
Ischemic stroke, n (%)	93(91.2)	54(91.5)	39(90.7)	0.884
Large-artery atherosclersis	63(67.7)	45(83.3)	18(46.2)	< 0.001
Small-artery occlusion	11(11.8)	3(5.6)	8(20.5)	0.060
Cardioembolism	1(1.1)	0	1(1.1)	0.419
Other determined etiology	4(4.3)	1(1.9)	3(7.7)	0.306
other undetermined etiology	14(15.1)	5(9.3)	9(23.1)	0.066
TIA, n (%)	16(15.7)	8(13.6)	8(18.6)	0.489
Complications of TIA and ischemic stroke	7(6.9)	3(5.1)	4(9.3)	0.663
Complications of ischemic and hemorrhagic stroke, n (%)	2(2.0)	2(3.4)	0	0.223
**Treatment**				
Antiplatelet drug, n (%)	95(93.1)	55(93.2)	40(93)	0.969
lipid-lowering drug, n (%)	92(90.2)	53(89.8)	39(90.7)	0.884
Endovascular treatment, n (%)	4(3.9)	3(5.1)	1(2.3)	0.636

### Retinal nerve fiber layer thickness in patients with and without intracranial atherosclerotic stenosis

**Fig. 2:**
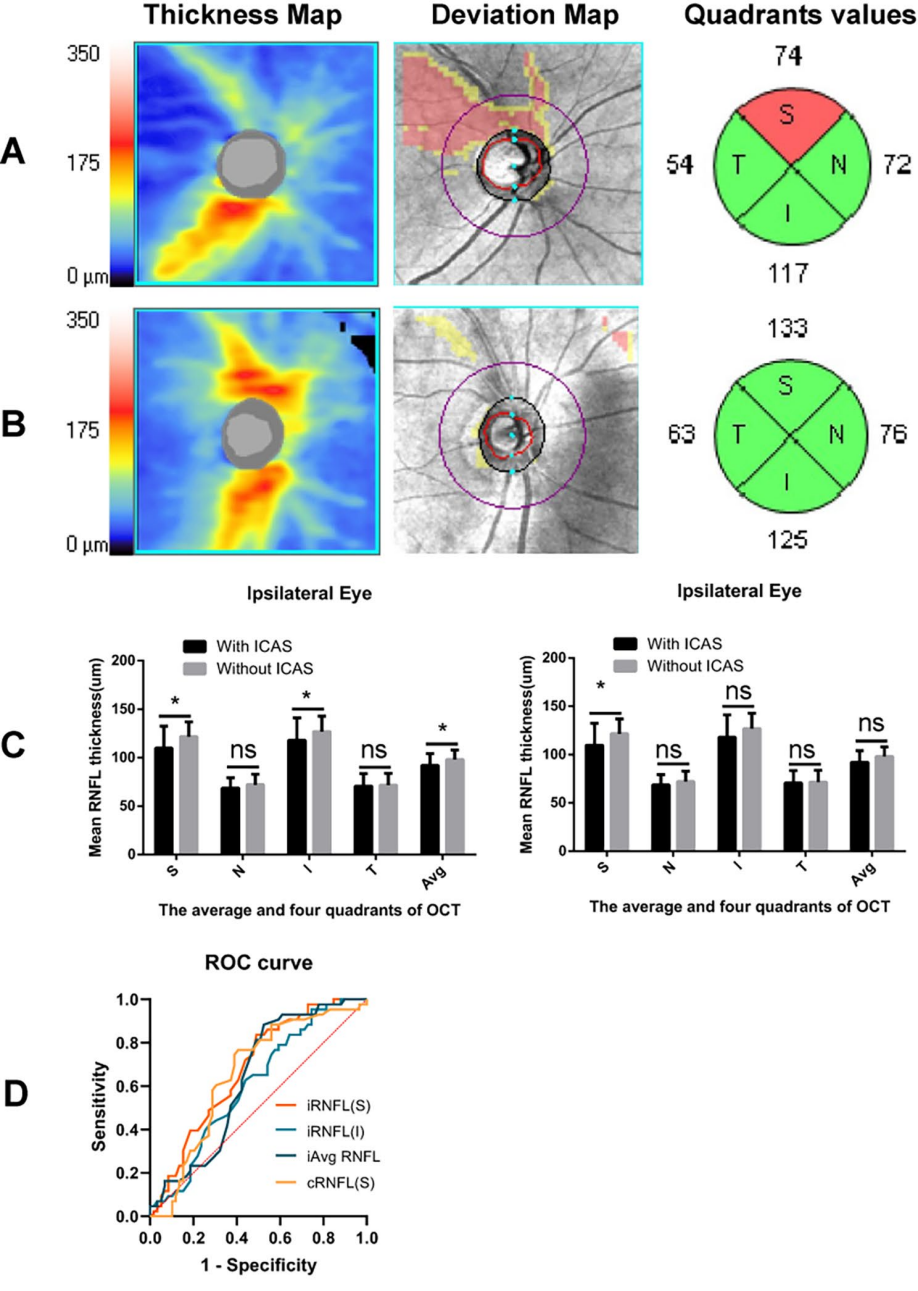
OCT images showed retinal nerve fiber layer (RNFL) thickness in eyes of patients with ICAS and without ICAS. A, B. Images of the thickness map, deviation map and quadrant values showed the superior quadrant of RNFL thinning in the ipsilateral eye of a representative case with ICAS compared with a case without ICAS. C. Comparisons of the average and four quadrants RNFL thickness between patients with and without ICAS in the ipsilateral eyes and contralateral eyes. *, p < 0.05; ns, no statistical difference. ICAS, intracranial atherosclerotic stenosis; S, superior; N, nasal; T, temporal; I, inferior; Avg, average. D. Receiver operator curves (ROC) showed the performance of retinal nerve fiber layer (RNFL) thickness in identifying intracranial atherosclerotic stenosis. The iRNFL(S), iRNFL(I) and iAvg RNFL represent the average and the superior, inferior quadrants of retinal nerve fiber layer thickness in the ipsilateral eyes, respectively. The cRNFL(S) represents the superior quadrants of retinal nerve fiber layer thickness in the contralateral eyes

Retinal nerve fiber layer thickness in patients with and without ICAS was shown in Table 2; Fig. 2. In the ipsilateral eyes, patients with ICAS were more likely to have thinner in the average pRNFL (mean [SD], 92 [12.1] vs. 98 [9.8], *p* = 0.008), the superior (mean [SD], 109.8 [22.6] vs. 121.6 [15.3], *p* = 0.004) and inferior (mean [SD], 118 [23.1] vs. 126.9 [15.9], *p* = 0.022) pRNFL thickness than patients without ICAS. In the contralateral eyes, the superior pRNFL thickness was reduced in patients with ICAS when compared with patients without ICAS (median [IQR], 118 [107–133]vs 129 [123–138], *p* = 0.007). However, there were no significant differences in the temporal and nasal quadrants of RNFL between patients with and without ICAS both in the ipsilateral eyes and contralateral eyes (*p* > 0.05 each).

**Table 2:** The retinal nerve fiber layer thickness of ipsilateral eyes and contralateral eyes in patients with and without ICAS

	All n = 102	With ICAS n = 59	Without ICAS n = 43	p value
**Ipsilateral eyes**				
RNFL(N)	70(64–76)	69(63–75)	70(65–76)	0.239
RNFL(S)	114.8(20.6)	109.8(22.6)	121.6(15.3)	0.004*
RNFL(T)	69(62–79)	69(62–77)	71(62–79)	0.564
RNFL(I)	121.7(20.8)	118(23.1)	126.9(15.9)	0.031*
Avg RNFL	94.5(11.5)	92(12.1)	98(9.8)	0.008*
**Contralateral eyes**				
RNFL(N)	66.5(12.4)	66.3(12.7)	66.8(12.1)	0.823
RNFL(S)	125(110-135.3)	118(107–133)	129(123–138)	0.007*
RNFL(T)	67(14.4)	65.7(13.3)	68.6(15.8)	0.318
RNFL(I)	119(107-134.3)	119(105–135)	119(110–133)	0.965
Avg RNFL	95(86.5-102.3)	94(83–101)	97(90–103)	0.173

Abbreviations:ICAS,intracranial atherosclerotic stenosis;RNFL, retinal nerve fiber layer;S, superior; N, nasal; T, temporal; I, inferior; Avg, average.*represents p < 0.05, there is significant difference between the two groups

### The association between retinal nerve fiber layer thickness and intracranial atherosclerotic stenosis

After spearman correlation analysis, patients with ICAS had a negative correlation with the superior (*p* = 0.002; ρ=-0.306) pRNFL and the average (*p* = 0.019; ρ=-0.232) pRNFL in the ipsilateral eyes, with the superior pRNFL (*p* = 0.007; ρ=-0.267) in the contralateral eyes. In univariate logistic analysis, the superior, inferior and average pRNFL in the ipsilateral eyes and the superior quadrant of pRNFL in the contralateral eyes were associated with ICAS. After multivariate analysis, only the superior quadrant of pRNFL in the ipsilateral eyes was significantly associated with ICAS (OR, 0.968; 95% CI,0.946–0.991; *p* = 0.006) (Table 3). The area under receiver operator curve for patients with ICAS versus patients without ICAS were 0.679 (95% CI, 0.576–0.782) for the superior pRNFL, 0.608 (95% CI,0.499–0.717) for the inferior pRNFL, 0.636 (0.528–0.744) for the average pRNFL in the ipsilateral eyes, and 0.656 (0.548–0.764) for the superior pRNFL in the contralateral eyes, respectively. The cut-off value of the superior quadrant of pRNFL in the ipsilateral eyes was 109.5 μm, of which the sensitivity and specificity were 50.8% and 83.7%, respectively. (Fig. 2)

**Table 3:** The relationship of retinal nerve fiber layer thickness and intracranial atherosclerosis stenosis

	nivariate analysis		Multivariate analysis	
Variables	OR (95%CI)	p value	OR (95%CI)	p value
**Ipsilateral eyes**				
RNFL(S)	0.968(0.946–0.991)	0.006*	0.968(0.946–0.991)	0.006*
RNFL(I)	0.977(0.957–0.998)	0.036*	-	-
Avg RNFL	0.952(0.916–0.989)	0.011*	-	-
**Contralateral eyes**				
RNFL(S)	0.982(0.961–1.004)	0.103		

Abbreviations:RNFL, retinal nerve fiber layer;S, superior; N, nasal; T, temporal; I, inferior; Avg, average.*represents p < 0.05 and has statistical significance

## Discussion

In this study, we demonstrated that peripapillary retinal nerve fiber layer thickness was associated with intracranial atherosclerotic stenosis. The superior, inferior and average RNFL were thinning in the ipsilateral eyes, while only the superior pRNFL was thinning in the contralateral eyes of patients with ICAS. As both eyes had changes in superior pRNFL, the changes may be related to some systemic conditions such as age. After multivariate analysis, the superior quadrant of pRNFL thickness in the ipsilateral eyes was significantly associated with ICAS. Furthermore, it had better performance in identifying ICAS than the other quadrants of pRNFL thickness. These findings suggest that retinal ganglion cells loss in retina may be in correlation with intracranial atherosclerotic stenosis.

Transneuronal retrograde degeneration (TRD) is neurons degeneration following the removal of the axons or terminals of their postsynaptic target cells, which has been observed in several neurons [7, 16]. An antegrade projection occurs in the eye from the retina to the primary visual cortex, while a retrograde projection occurs from the primary visual cortex to the retina [17]. Non-human primate studies and human eyes researches have reported that TRD occurred in the retinal ganglion cells after occipital lobectomy [18–21]. The retinal nerve fiber layer is constituted of the axons of the RGCs and frequently used to assess ganglion cell changes [16]. Park et al. have confirmed that the superior, inferior, nasal and temporal quadrants of retinal nerve fiber layer thickness thinning in patients with cerebral infarction, providing evidence for TRD of the RGCs, particularly more prominent in the nasal RNFL of the contralateral side and in the temporal RNFL of the ipsilateral side of cerebral damage [17]. R. Anjos et al. have found a peripapillary RNFL and a macular ganglion cell layer thinning in both eyes of patients with posterior cerebral artery ischemic lesion, suggesting that TRD may play a role in the physiopathology of lesions of the posterior visual pathway [22]. In this study, we found that the superior, inferior quadrants and the average of peripapillary RNFL in the ipsilateral eyes were associated with ICAS. However, the temporal and nasal quadrants of pRNFL were not associated with ICAS both in the ipsilateral eyes and contralateral eyes. Moreover, the average and the four quadrants of the pRNFL in the contralateral eyes of patients were also not associated with ICAS. The appearance may be a unique pattern of RGCs loss presentation with intracranial atherosclerotic stenosis and may be regarded as a biomarker for identifying the presence of ICAS. Definitely, further studies are needed to confirm and validate the applicability in clinical practice. The optic nerve fibers of RGCs in the nasal hemi retina cross at the optic chiasm, whereas those in the temporal hemiretina do not, and the superior, inferior, and temple peripapillary retina can allow both crossed and uncrossed fibers to travel to the optic disc [17]. Different distributions of crossed and uncrossed fibers in four quadrants may lead to this situation. Further studies will be required to investigate the mechanism.

Middle cerebral artery occlusion (MCAO) is a common model used to study cerebral ischemia in rodents [23]. MCAO can block both the ophthalmic artery and the middle cerebral artery, causing both cerebral and retinal ischemia duo to the proximity of the anatomical position [23]. Some animal studies have confirmed that retinal ischemia and dysfunction at 48 h or even 9 days post-MCAO [23, 24]. Besides, clinical studies have reported that ICAS was associated with retinal vascular changes such as enhanced arteriolar light reflex and retinal diameter variation [25, 26]. A study showed that retinal vascular changes were associated with large-artery stroke, suggesting that retinal vessels structural changes may result from downstream effects of large artery pathology in the retinal and cerebral circulations [27, 28]. Besides, localized RNFL defects was associated with acute ischemic stroke after adjustment for systemic and ocular factors [29, 30]. In our study, we found that ischemic stroke induced by large-artery atherosclerosis had a larger proportion in patients with ICAS than those without ICAS. Intracranial atherosclerotic stenosis induced stroke may be related to artery-to-artery embolism, plaque extension over small perforator artery ostia hypoperfusion, or combined mechanisms [31]. Thus, we speculated that stroke-related retinal nerve injury may be not only linked to TRD, but also to hemodynamic changes caused by ICAS.

Optical coherence tomography (OCT) is a reliable noninvasive retinal imaging technique, providing high-resolution imaging based on the principle of low coherence interferometry that enables remarkable advances in assessing RGC axons by quantifying cross-sectional imaging of peripapillary retinal nerve fiber layer damage [1, 30, 32]. OCT can provide retinal sectional images in vivo similar to pathological section of histology study and has great advantages including simple, rapid, noninvasive, inexpensive, reproducible, and real-time display [30, 33]. It is widely used to assess RGC neuronal abnormalities in other neurological diseases such as dementia [3] and stroke [7]. Thus, OCT may be a feasible and promising tool by quantifying the superior quadrant of RNFL as a biomarker to identify the presence of ICAS in future.

There were some limitations in our study. First, this study was a single center cross-sectional study and may not be appliable to other populations. A multicenter, large scale based on population longitudinal study are needed to validate the association. Second, the present findings may not explain clearly the unique appearance of the RNFL thickness thinning inconsistency in four quadrants and the mechanism of RGC loss in patients with ICAS. Third, few patients with immature cataracts were included in the study, which might affect the OCT RNFL results [34]. Finally, further research is needed to investigate the relationship between ICAS and the macular ganglion cell layer thickness or macular volume.

In conclusion, we demonstrated that the superior quadrant of peripapillary retinal nerve fiber layer thickness in the ipsilateral eyes was significantly associated with intracranial atherosclerotic stenosis. It showed moderate performance in identifying ICAS. Besides, intracranial atherosclerotic stenosis may be an underlaying pathogenesis for retinal nerve injury after cerebrovascular events occurred. Further studies are still needed to explore the relation in detail.

## Data Availability

The data that support the findings of this study are available from the corresponding author on reasonable request.
